# Information Contained in Molecular Motion

**DOI:** 10.3390/e21111052

**Published:** 2019-10-28

**Authors:** J Gerhard Müller

**Affiliations:** Department of Applied Sciences and Mechatronics, Munich University of Applied Sciences, D-80335 Munich, Germany; gerhard.mueller@hm.edu

**Keywords:** information, entropy, molecular motion, measurement, information gain, information erasure

## Abstract

The equivalence between information and entropy is used to interpret the entropy of a molecular gas as missing information about its internal state of motion. Our considerations show that thermodynamic information is principally composed of two parts which continually change in the course of gas-kinetic collisions. While the first part relates to energy carried by the individual molecules in the form of kinetic energy and in internal excitations, the second relates to information concerned with the location of the molecules within their own mean-free volumes. It is shown that this second kind of information is generated in gas-kinetic collisions and rapidly deteriorated and lost by quantum mechanical dispersion until it is re-gained in follow-on collisions. It is proposed that gas-kinetic collisions can be regarded as measurement processes in which information is continually gained, deteriorated and erased. As these processes occur naturally without any human intervention, it is argued that thermodynamic information—like entropy—fully qualifies as an objective physical quantity.

## 1. Introduction

The concept of entropy was introduced by Clausius [1] in the nineteenth century into the evolving science of thermodynamics. In this context a change in entropy S was defined as the amount of heat, $\delta Q_{rev}$, reversibly exchanged between two macroscopic bodies maintained at an absolute temperature T:

(1) ΔS=δQrevT

Later, this macroscopically measurable quantity was explained in the context of statistical mechanics as molecular disorder contained in a macroscopic body. According to Boltzmann [2], entropy is related to the number W of microscopic states of motion which are consistent with the limited macroscopic knowledge about the molecular system:

(2) $$S=k_{B}\ln\left(W\right),\;k_{B}=8.62*10^{5}\frac{eV}{K}$$

One of the most striking facts about entropy is its tendency to increase in an irreversible manner within an isolated system. This tendency was interpreted early on as arising from the statistics of motion of large numbers of particles rather than from any irreversibility in the underlying laws of motion of the individual molecules themselves. Considering the intrinsic reversibility of the basic mechanical laws it was argued by Maxwell [3] that a being *“whose faculties are so sharpened that it can follow the course of individual molecules”* should be able to operate a trap door, inserted into a vessel homogeneously filled with gas, to produce either temperature or pressure differences inside the gas that would allow mechanical work to be extracted from a single macroscopic heat reservoir, maintained at a finite temperature T. Such a gain in energy, clearly, would be in sharp contrast with the postulate imposed by the second law of thermodynamics and thus create a paradox. Since its invention in 1871, Maxwell’s demon paradox has fascinated researchers up to the present day. A good coverage of the historical developments can be found in the books of Rex and Leff [4,5].

A decisive step forward in the analysis of Maxwell’s demons´ paradox was made by Leo Szilard in 1929 [6]. Considering the motion of a single atom, enclosed in a finite volume V and in contact with a heat reservoir of temperature T, Szilard showed that knowledge about the location of the atom can be used to extract mechanical work from a single-molecule gas. This example showed that work extraction from a thermal reservoir is not for free but that it has to be paid for in terms of a new “currency”, called information. The other important achievement was that Szilard’s analysis established a connection between the physical concepts of entropy and abstract information, showing that 1 bit of abstract information corresponds to $k_{B}\ln\left(2\right)$ units of entropy. This definition of a bit as the unit of information is generally regarded as the starting point of modern information theory [7,8]. 

A further important step in the analysis of Maxwell’s demon paradox was the realization that the information gained in a physical measurement is burdened with an energy cost that overwhelms the energy gained in operating a Maxwell’s demon device. Brillouin’s initial analysis [9], which claimed that this energy cost is associated with the measurement process itself was later challenged by arguments that reversible measurements might in principle be conceivable [4,5]. Even later it was argued that operating a Maxwell’s demon device entails storage of the information intermittently obtained, and that the energy cost of information ultimately needs to be paid when this information is erased again. It was argued that this latter step of erasure is unavoidable to arrive at a fully cyclic process of information gain and work extraction and that therefore the energy cost of information erasure is a more inescapable necessity than the energy cost of measurement and information gain. This latter resolution of Maxwell’s demons’ paradox became known as Landauer’s principle [10]. Brillouin and Landauer both agree in that ultimately an energy of at least $W=k_{B}T\ln\left(2\right)$ needs to be expended to gain one bit of information and to ultimately conserve the validity of the second law.

Following discussions of Maxwell’s demon’s paradox, it can be observed that some authors express discomfort with the idea of thermodynamic information as the processes of gaining, receiving and maintaining information through measurement either explicitly or implicitly assume the involvement of intelligent beings [3,4,5,6]. It is argued that such an involvement introduces an element of subjectivity into the definition of entropy, which is unacceptable if entropy is to be considered as an element of physical reality that exists independent of the existence of human beings and of human observation [11,12]. 

In the present paper we consider the entropy of a molecular gas and interpret it as information carried by its molecular constituents. In §2 we consider the Sackur-Tetrode equation for the entropy of an ideal gas [13] consisting of elastic hard spheres of very small size, but without any internal structure. Transforming this equation to information units, the fact of ignorance about the molecular motion on the side of an outside observer is turned into a more positive statement about information that is carried with the mechanical motion of the gas molecules itself, but which remains unknown to the outside observer. With the help of the Sackur-Tetrode equation we arrive at the result that the individual molecules inside an ideal gas carry two kinds of information, the first one being related to the kinetic energy of molecules carried with their center-of-mass motion and the second to information related to the positions of gas molecules within their own mean-free volumes. With the Sackur-Tetrode equation being a result of equilibrium statistical mechanics, this equation provides a static and time-averaged picture of an ideal gas in which all molecules are treated alike, always moving with the same kinetic energy and always occupying the same effective space, independent of time. In §3 we therefore go beyond the limits of the Sackur-Tetrode equation and turn to a discussion of gas-kinetic collisions. There, it is argued that with gas-kinetic collisions taking place, the individual pieces of molecular information must depend on time with energy and initial localization changing discontinuously during gas-kinetic collisions and molecular localization changing continuously during times in between successive collisions. This latter effect is the most interesting one as it shows that the information about the particle location is initially determined by the energy transfers that have taken place during a first collision and that this kind of information deteriorates and finally gets lost as the molecules travel through their respective mean-free paths and as these become likely to undergo follow-on collisions. In §4, it is then argued that these repeated gains and losses in molecular information resemble measurement processes in which information is repeatedly gained, deteriorated, erased and finally updated again as gas-kinetic collisions occur. Considering two thought-experiments on a single-molecule gas, it is shown that the proposed continuous variation of molecular information is in principle experimentally accessible when the experiment is performed in a supervised manner but inaccessible otherwise. Realizing that in both experiments the basic processes of information gain and information erasure follow the same kind of physics, it is argued that both processes do not require the intervention of intelligent beings and that therefore entropy and thermodynamic information alike satisfy the requirement that physical entities should exist independent of the existence and observation of intelligent beings. In §5, we extend our considerations to molecules with a more complicated internal structure which allows them to store energy in the form of molecular rotations, vibrations and electronic excitations and to exchange such pieces of energy during gas-kinetic collisions. In §6, we summarize our results and provide an outlook towards possible future research. 

## 2. Entropy of an Ideal Gas

**Figure 1:**
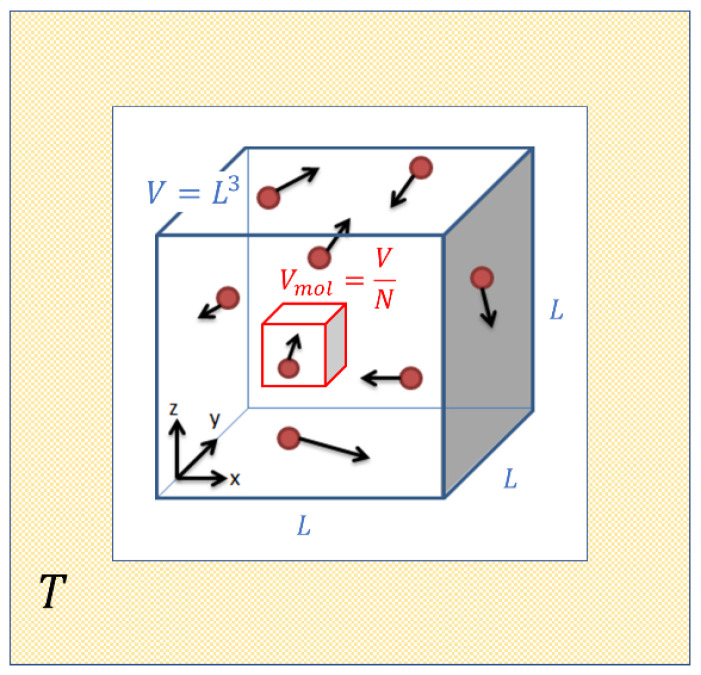
Sketch of the molecular motion inside an ideal gas enclosed in a volume V and maintained at a temperature T.

Ideal gases are mental constructs which replace the complex molecules in real gases by simple elastic hard spheres of very small size, which collide from time to time and which thereby exchange energy and momentum. As is well known, this strongly simplified picture approximates the behavior of real gases in the limit of high temperature and low pressure. Such a situation is visualized in Figure 1 which assumes that N molecules are contained in a box of volume $V=L^{3}$ which in turn is embedded in a heat reservoir of temperature T. As the molecules move inside this box they trace out, on average, a volume $V_{mol}=\left(V/N\right)$ before suffering a gas-kinetic collision.

The entropy of such a gas is experimentally accessible by measuring the amount of heat that needs to be fed into the volume V as the gas is heated up from very low temperatures up to the reservoir temperature T:

(3) $$S_{gas}\left(T,V,N\right)=V\int_{0}^{T}\frac{c_{V}\left(\theta \right)}{\theta }d\theta$$

In this equation $c_{V}\left(T\right)$ is the heat capacity at constant volume for a gas containing ngas=NV molecules per unit volume. Once the reservoir temperature has been stabilized, the quantity $S_{gas}\left(T,V,N\right)$ has been fixed and from then on represents a quantitative measure of ignorance that exists on the side of the outside observer with regard to the details of the molecular motion inside the volume V. 

With the help of equilibrium statistical mechanics, the entropy $S_{gas}\left(T,V,N\right)$ can be related to those three parameters N,V and T that are known and under control of the outside observer:

(4) $$S_{gas}\left(T,V,N\right)=N\left\{\frac{5}{2}k_{B}+k_{B}ln\left[\frac{V_{mol}}{V_{Q}\left(T,M\right)}\right]\right\}$$

This result, which has originally been derived by Sakur and Tetrode [13] involves as a key parameter the quantum volume:

(5) $$V_{Q}\left(T,M\right)={\left(\frac{2\pi \hbar^{2}}{Mk_{B}T}\right)}^{\frac{3}{2}}$$

which up to a factor of the order of unity is the third power of the thermal de-Broglie wavelength of the moving particles:

(6) $$\lambda_{th}\left(T,M\right)=\frac{\hbar }{\sqrt{3Mk_{B}T}}$$

Here, $p_{th}=$ $\sqrt{3Mk_{B}T}$ stands for the momentum of a molecule of mass M moving with a kinetic energy of $E_{th}=\frac{3}{2}k_{B}T$ and ℏ for the reduced Planck’s constant.

The statement of ignorance, expressed by the function $S_{gas}\left(T,V,N\right)$, can be turned into more positive fashion by converting $S_{gas}\left(T,V,N\right)$ to information units:

(7) $$I_{gas}\left(T,V,N\right)=\frac{1}{k_{B}ln\left(2\right)}S_{gas}\left(T,V,N\right).$$

In this latter form, the function $I_{gas}\left(T,V,N\right)$ represents an amount of information that is contained in the molecular motion of the gas and that remains unknown to the outside observer whose knowledge is confined to the three state parameters T,V,N. Further, considering that the entropy $S_{gas}\left(T,V,N\right)$ can be expressed in the form $S_{gas}\left(T,V,N\right)=Ns_{mol}\left(T,V,N\right)$, the molar entropies, $s_{mol}\left(T,V,N\right)$, can be interpreted as information that, on average, is missing to an outside observer with regard to each molecule inside the gas:

(8) $$i_{mol}\left(T,V,N\right)=\frac{1}{\ln\left(2\right)}\left\{\frac{5}{2}+ln\left[\frac{V_{mol}}{V_{Q}\left(T,M\right)}\right]\right\}$$

The latter equation shows that the molecular information principally consists of two parts, which can be interpreted as information, $i_{ccm}\left(T,V,N\right)$, that is missing with regard to the center-of-mass motion of each molecule and $i_{loc}\left(T,V,N\right)$ as information that is missing with regard to its location within its own mean-free volume:

(9) $$i_{mol}\left(T,V,N\right)=i_{ccm}\left(T,V,N\right)+i_{loc}\left(T,V,N\right).$$

These latter assignments can be rationalized as follows: turning to $i_{cmm}$ first, we remember that in an ideal gas each particle, on average, carries an energy Eav=12kBT per degree of freedom f, $i_{cmm}$ therefore can be expressed as:

(10) $$i_{cmm}\left(T,V,N\right)=\frac{1}{ln\left(2\right)}\frac{fE_{av}}{k_{B}T}$$

The number of f=5 degrees of freedom can be rationalized by considering that each particle enjoys 3 degrees of freedom regarding motion along the three orthogonal directions in space and two further degrees of freedom as each particle works against the constant-pressure background generated by the N−1 companion molecules inside the gas [13]. 

The interpretation of the second term, $i_{loc}$, is straight-forward if one considers that $V_{Q}\left(T,M\right)$ is the effective size of a molecule moving with its mean thermal energy through the gas. Localization of the molecule within its mean-free volume reduces the number n of possibilities of finding it somewhere inside this volume from $n_{initial}=\frac{V_{mol}}{V_{Q}\left(T,M\right)}$ to $n_{final}=1$. The associated gain in information is then:

(11) $$i_{loc}\left(T,V,N\right)=log_{2}\left[\frac{V_{mol}}{V_{Q}\left(T,M\right)}\right]=\frac{1}{ln\left(2\right)}ln\left[\frac{V_{mol}}{V_{Q}\left(T,M\right)}\right]$$

## 3. Gas-kinetic Interactions

Being a result of equilibrium statistical mechanics, the Sackur-Tetrode equation (Equations (4) and (5)) provides a static and time-averaged picture of an ideal gas in which each molecule is treated alike. In particular, it suggests that each molecule is moving with the same kinetic energy and that each molecule occupies the same volume $V_{Q}\left(T,M\right)$, independent of time. 

This static picture sharply contrasts with the situation in real gases: in a real gas under normal temperature-pressure (NTP) conditions, the average distance between molecules is in the order of $d_{av}\approx 5\times 10^{-5}$ cm. Depending on the molecular mass, the molecules inside such a gas travel these distances with speeds around $v_{th}\approx 5\times 10^{4}$ cm/s and thus, on average, collide with each other after times $\tau_{coll}\approx 10^{-9}$ s. In these interactions energy and momentum is exchanged between the collision partners and a Maxwellian velocity distribution is established. 

Allowing for gas-kinetic interactions, kinetic energy is constantly re-shuffled among gas molecules with an average rate of $\nu_{coll}=1/\tau_{coll}$ and collision partners become localized as energy is transferred between them. In such a situation the pieces of information relating to the different gas molecules become molecule-specific and time-dependent:

(12) $${i_{mol}}_{i,j}\left(t\right)={i_{cmm}}_{i,j}+{i_{loc}}_{i,j}\left(t\right)$$

In this equation the index i enumerates the individual molecules (i=1…N) and j the particular collisions; t, finally, is the time between successive collisions j and j+1. As before, the ${i_{cmm}}_{i,j}$ and ${i_{loc}}_{i,j}$ take on the forms:

(13) $${i_{cmm}}_{i,j}=\frac{1}{\ln\left(2\right)}\frac{{E_{kin}}_{i,j}}{k_{B}T}$$

(14) $${i_{loc}}_{i,j}=\frac{1}{ln\left(2\right)}ln\left[\frac{V_{mol}}{{V_{Q}}_{i,j}}\right].$$

In this latter equation, the ${V_{Q}}_{i,j}$ are the quantum volumes of molecules moving with energies ${E_{kin}}_{i,j}$. As de-Broglie wavelengths depend on particle momenta and as shown in more detail in the Appendix A, the ${V_{Q}}_{i,j}$ exhibit an energy dependence according to:

(15) $${V_{Q}}_{i,j}={\left(\frac{2\pi^{2}\hbar^{2}}{M{E_{kin}}_{i,j}}\right)}^{3/2}$$

A particularly interesting aspect is that the ${i_{loc}}_{i,j}$ also exhibit a continuous dependence on time during those phases in which the individual molecules move freely in between successive collisions:

(16) $${i_{loc}}_{i,j}={i_{loc}}_{i,j}\left(t\right)$$

**Figure 2:**
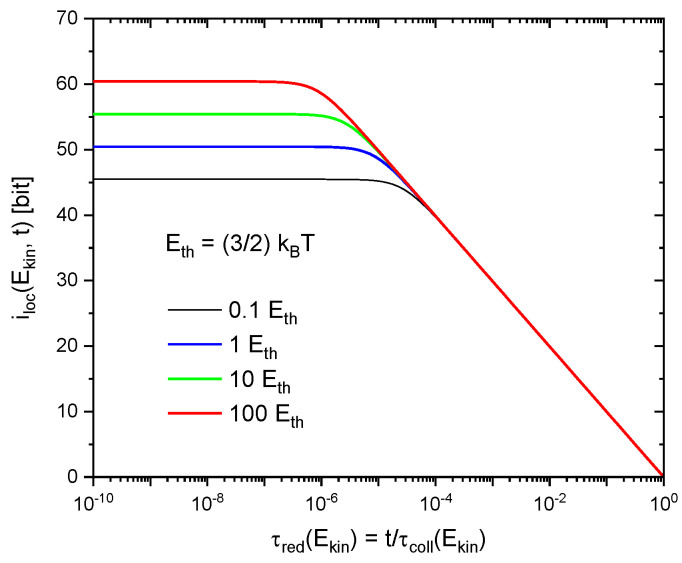
Variation of $i_{loc}\left(E_{kin},t\right)$ (Equation A12) during successive gas-kinetic collisions as a function of reduced time $\tau_{red}=t/\tau_{coll}$, where $\tau_{coll}=\tau_{coll}\left(E_{kin}\right)$ represents the energy-dependent collision time at which follow-on collisions are likely to take place. The individual curves denote solutions for increasing molecular kinetic energies in units of $E_{th}$. Molecular parameters correspond to normal air (N_2_, O_2_).

This latter effect arises from the phenomenon of quantum-mechanical dispersion which causes the wave functions of the molecules to spread out in space as these are moving freely through empty space in between successive collisions. As shown in the Appendix A, the 3d molecular wave packets initially become localized up to the constraints imposed by the uncertainty relationships and then rapidly spread out to volumes ${V_{Q}}_{i,j}$ comparable to the average mean-free volume $V_{mol}$ in the gas at times at which the molecules become likely to undergo follow-on collisions. As a consequence, all ${i_{loc}}_{i,j}$, independent of kinetic energy, tend to zero immediately before follow-on collisions are likely to occur. This latter effect is illustrated in Figure 2 for functions ${i_{loc}}_{i,j}\left(t\right)$, appropriate to different values of ${E_{kin}}_{i,j}$ gained in preceding collisions. In order to illustrate this energy-independence, all curves in Figure 2 have been plotted as functions of their respective reduced times

(17) $$\tau_{red}\left(E_{kin}\right)=t/\tau_{coll\;}\left(E_{kin}\right)$$

with $\tau_{coll}\left(E_{kin}\right)$ standing for the mean-free time of molecules moving with kinetic energy $E_{kin}$ in between successive collisions:

(18) $$\tau_{coll}\left(E_{kin}\right)=d_{av}\sqrt{\frac{M}{2E_{kin}}}$$

With this scaling applied, the results in Figure 2 show that, during gas-kinetic collisions, the individual molecules repeatedly gain and loose information with regard to their localization as these are moving on in between successive collisions. 

## 4. Supervised and Unsupervised Measurement Processes

Information about physical phenomena is conventionally gained in measurements designed and performed by human beings. With this background in mind, the changes in $i_{loc}\left(E_{kin},t\right)$, displayed in Figure 2, can be regarded as measurement processes in which information is intermittently gained, deteriorated, lost and finally updated again as gas-kinetic collisions occur. At this point, it is relevant to note that we defined the functions $i_{mol}\left(T,V,N\right)$ (Equations (8) and (9)) and their time-dependent extrapolations (Equations (12)–(16)) as information carried by the individual molecules, which otherwise remains unknown to outside observers. Remembering this fact of unobservability, it is clear that these naturally occurring measurements proceed in an un-supervised manner, in sharp contrast to conventional ones. 

In this section, we consider two thought experiments, performed on single-molecule gases, which are carried out in a supervised and in an un-supervised mode. These considerations show that the pieces of information ${i_{mol}}_{i,j}\left(t\right)$ are principally accessible in the supervised mode but remain undetectable otherwise. In particular, we show that by performing many measurements in the supervised mode the Maxwellian velocity distribution can be re-established while in the unsupervised mode only learned guesses concerning the molecular motion are possible and that these lead back to the time-averaged Sackur-Tetrode result $i_{mol}\left(T,V,N\right)$ (Equation (8)).

**Figure 3:**
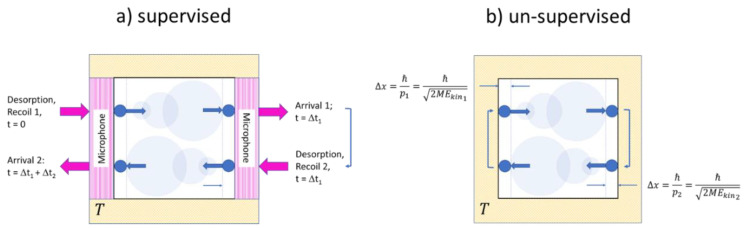
Sketch of molecular motion inside single-molecule gases designed to allow for supervised (**a**) and un-supervised (**b**) observations. Quantum-mechanical dispersion of the molecular wavefunctions is indicated by circles of increasing size. Forward and backward travels have been vertically displaced for ease of presentation.

Both experiments are sketched in Figure 3. In both experiments the outside observer knows from their preparation that a single molecule of mass M has been filled into a volume $V=L^{3}$ and that this volume is then maintained at a temperature T. Both experiments differ in the way that two of the walls in the supervised version (Figure 3a) consist of ultra-sensitive microphones which are able to detect wall-molecule and molecule-wall interactions, while in the unsupervised version (Figure 3b) all walls are simply macroscopic heat reservoirs without any sensory capabilities. Concerning the internal molecular motion, we assume that in both gases the molecule is initially adsorbed on the left-hand wall and that it takes off from the wall with a momentum $p_{lr}=\sqrt{2ME_{kin\_lr}}$ with Ekin_lr≫Eth=32kBT, thus enabling it to move horizontally towards the right-hand wall, which will be reached in a time τlr=LM2Ekin_lr. Both upon leaving the left-hand wall and arriving at the right-hand wall, signals will be generated which allows the outside observer to determine the transit time $\tau_{lr}$ and to calculate the kinetic energy and the linear momentum of the molecule. Further knowing that the molecule is adequately described by a 3d quantum-mechanical wave packet, its initial volume upon take-off and its dispersion during its travel to the right-hand wall can be ascertained. Similar arguments apply to the backward travel shown in the lower section of Figure 3a where the molecule might have started its journey back with a different momentum $p_{rl}=\sqrt{2ME_{kin\_rl}}$. In brief, the dynamics displayed in Figure 3a can be reconstructed with a simple knowledge of basic mechanics and quantum mechanics. In case this experiment is repeated, the Maxwellian velocity distribution can be re-established. 

With the same kind of molecular motion, the situation in the un-supervised experiment (Figure 3b is a very different one: with the knowledge of the outside observer being limited to the three state parameters T,V,N, the outside observer can only guess that the molecule is moving with its most probable energy Eth=32kBT. With this assumption, the proper guess for the quantum volume directly after a collision is $V_{Q}\left(T,M\right)$ (Equation (5)). With the timing signals of the supervised experiment not being available, the outside observer cannot reconstruct the time evolution of the quantum volume and thus not reconstruct the function ${i_{mol}}_{i,j}\left(t\right)$ that would appropriately describe the molecule on its travel between both walls. With this timing information not being available, the outside observer therefore remains with the maximum uncertainty concerning the molecule’s position inside the box, which is given by $V_{Q}\left(T,M\right)$ (Equation (5)). With this uncertainty in mind, the function $i_{loc}\left(T,V,N\right)$ (Equation (11)) is retained. With all these learned guesses, the outside observer finally arrives at:

(19) $$i_{mol}\left(T,V,N\right)=\frac{1}{\ln\left(2\right)}\left\{\frac{3}{2}+ln\left[\frac{V_{mol}}{V_{Q}\left(T,M\right)}\right]\right\}$$

which is exactly the Sackur-Tetrode equation (Equation (5)) when the correct limit to N→1 molecules is taken. 

Overall, both sketches show that the physics underlying supervised and unsupervised measurements is basically the same, involving particle-wall interactions, wave packet localization, wave dispersion and wave collapse during forward and backward travels. Not detecting any fundamental differences between supervised and un-supervised measurements, it is concluded that intelligent observers are not necessary to initiate and carry out processes of information gain and of information erasure. Thus, thermodynamic information, like entropy both qualify as physical quantities, independent of the existence and observation by human observers.

## 5. Gases with Internal Degrees of Freedom

So far, we have been dealing with ideal gases which are conceived as consisting of elastic hard spheres without any internal degrees of freedom. Arguing within the constraints of this model we have arrived at the conclusion that the information relating to the center-of-mass motion of a particular molecule is valued by the ratio of its kinetic energy $E_{kin}$ relative to the average thermal energy $k_{B}T$ inside the reservoir formed by the N−1 background molecules. In this section we show that the validity of Equation (13) is not limited to the kinetic energy of the center-of-mass motion alone, but that it is of more general validity, extending to other forms of energy as well. 

**Figure 4:**
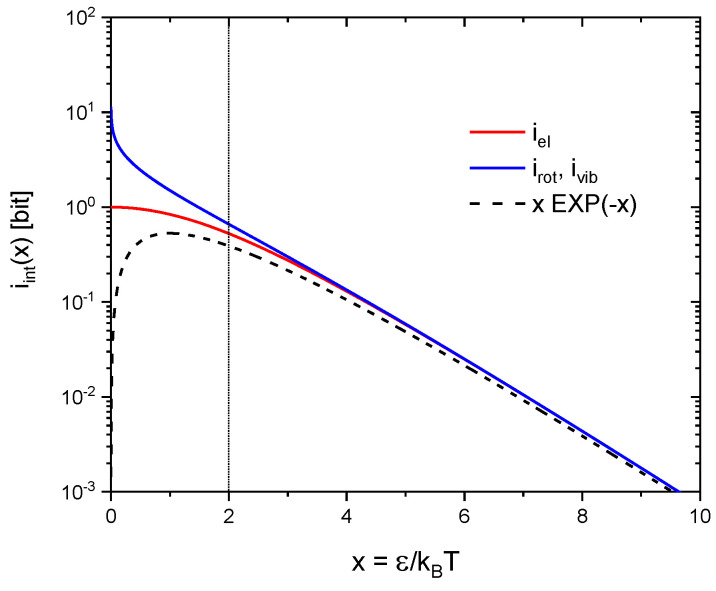
Molecular entropies as a function of the reduced inverse temperature $x=\varepsilon /k_{B}T$ as plotted in information units: (blue) rotations and vibrations following Bose-Einstein and (red) electronic excitations following Fermi-Dirac statistics. The dashed black line denotes the low-temperature approximation to both kinds of entropies, i.e., to excitation energies $\varepsilon \gg k_{B}T$.

In addition to moving along the three orthogonal directions in geometrical space, real molecules also enjoy internal degrees of freedom. In gas-kinetic interactions the energy stored in such internal degrees of freedom can be exchanged with collision partners and re-appear as kinetic energy as the collision partners move away from their sites of impact. Alternatively, kinetic energy may become fully or partly transferred into internal degrees of freedom as gas-kinetic collisions occur. In statistical mechanics the entropies of molecular rotations, vibrations and electronic excitations can be obtained from the simplified models of rigid rotators, linear oscillators and electronic two-level systems [13] with the first two obeying Bose-Einstein and the latter Fermi-Dirac statistics. For the convenience of the reader these molecular entropies are listed in Table 1 and their temperature dependencies are displayed in Figure 4. From this latter figure it can be seen that for excitation energies $\varepsilon >2k_{B}T$ all entropies reasonably well follow temperature dependencies of the form.

(20) $$S_{int}\left(\varepsilon ,T\right)=\left(\frac{\varepsilon }{k_{B}T}\right)\exp\left(-\frac{\varepsilon }{k_{B}T}\right)$$

In terms of information units these entropies correspond to information contents of:

(21) $$i_{int}\left(\varepsilon ,T\right)=\frac{1}{ln\left(2\right)}\left(\frac{\varepsilon }{k_{B}T}\right)exp\left(-\frac{\varepsilon }{k_{B}T}\right)$$

Equation (21) can be interpreted in the way that a two-level system actually excited with an energy $\varepsilon >2k_{B}T$ carries potential information in accordance with Equation (13). The additional exponential term arises from the fact that in N gas-kinetic interactions only $n\approx Nexp\left(-\frac{\varepsilon }{k_{B}T}\right)$ systems are expected to be encountered in their excited states. Significant differences in the statistical behavior of rotations, vibrations and electronic excitations only shine up when the mean thermal energy $k_{B}T$ becomes large compared to the respective quantum energies ($k_{B}T\gg \varepsilon$). In electronic two-level systems equal numbers of excited and non-excited electronic systems will be encountered at very high temperatures. The information gain upon interaction therefore saturates at 1 bit per interaction, i.e., in the decision of a simple alternative. In contrast, increasing numbers of excitations can assemble in rotational and vibrational two-level systems as the mean thermal energy is raised beyond $k_{B}T>\varepsilon$. The information gain upon interaction, therefore, can raise far beyond the Fermi-Dirac limit of 1 bit.

## 6. Conclusions and Outlook

The equivalence between information and entropy has been applied to the entropy of a molecular gas to interpret its entropy as information that is carried with its molecular constituents but missing to outside observers. The discussion has shown that the individual molecules inside the gas carry two kinds of information, which continually change as gas-kinetic collisions take place. The first part relates to energy that is stored in the external and internal excitations of the gas molecules, i.e., in their center-of-mass motion and in molecular rotations, vibrations and electronic excitations. All these forms of energies can be interchanged in gas-kinetic collisions and cause the wave functions of collision partners to become sharply localized to volumes much smaller than the average mean-free volume $V_{mol}$ of the molecules inside the gas. As the resulting localization is determined by the energies ε, carried in the external and internal excitations, these first forms of information can be summarized under the name “potential information”:

(22) $$i_{pot}\left(\varepsilon ,T\right)=\frac{1}{ln\left(2\right)}\frac{\varepsilon }{k_{B}T}\;\varepsilon \gg k_{B}T.$$

The second form of information relates to the positions of gas molecules within their own mean-free volumes. Being the result of transferred energies, this second form of information can be called “realized information”:

(23) $$i_{real}\left(\varepsilon ,T\right)=\frac{1}{ln\left(2\right)}\left[\frac{V_{mol}}{V_{Q}\left(\varepsilon ,T\right)}\right]$$

This second form of information contains as a key parameter the quantum volume $V_{Q}\left(\varepsilon ,\;T\right)$ which measures the extent of the molecular wave functions of the collision partners. As due to the effects of quantum-mechanical dispersion, the size of such wave packets changes as molecules move through their own mean-free volumes, the pieces of realized information are initially high directly after collisions and rapidly deteriorate and finally vanish shortly before follow-on collisions are likely to occur. 

It is proposed that these repeated changes in realized information in between successive collisions can be regarded as “measurement” processes in which information is continually generated, dispersed and erased without any human intervention. Extending the concept of “measurement” to naturally occurring physical interaction processes removes the need of “intelligence” in the processes of gaining and erasing information. In this sense it is further proposed that, entropy and thermodynamic information both fully qualify as objective physical entities, completely independent of human observation and interaction.

Before concluding we want to point out that the concepts of potential and realized information also appear to be fruitful in considering supervised measurement processes involving human observers. As an example, we mention the process of particle detection in man-made detectors. In such technical devices the potential information carried with the particle is converted into realized information by dissipating the particle energy $E_{p}$ in a piece of macroscopic matter maintained at a temperature $T_{d}$. In order to be useful as information-generating devices, such devices are constructed in a way that during the process of energy dissipation a macroscopically observable output signal is created, which can be taken as proof that a particle has interacted with the device at a certain time $t_{d}$ and at the location $x_{d}$ of the detector device. Such output signals represent events localized at space-time coordinates $\left(t_{d},x_{d}\right)$ which are endowed with a certain significance $I_{d}\left(E_{p},T_{d}\right)$ which depends on the signal-to-noise ratio, SN, realized under the specific conditions of detection. We will show in a forthcoming paper [14] that the information gain in detection:

(24) $$I_{d}\left(E_{p},T_{d}\right)=\frac{1}{ln\left(2\right)}ln\left(SN\right),$$

always remains smaller than the potential information, carried by the particle relative to the detector device:

(25) $$I_{d}\left(E_{p},T_{d}\right)<I_{pot}\left(E_{p},T_{d}\right)=\frac{1}{ln\left(2\right)}\frac{E_{p}}{k_{B}T_{d}}$$

This latter equation shows that a technical device only incompletely recovers the potential information that has been intrinsically carried with the particle.

With an observable event having been generated, the possibility exists that this event might be observed by observers moving in different frames of reference. In these different frames both the particle energy $E_{p}$ and the temperature of the detector $T_{d}$ will look different to the different observers. As a consequence, the different observers will in general associate different levels of significance to one and the same event:

(26) $$I_{d}\left(E_{p},T_{d}\right)\neq I_{d}\left(E_{p}{}^{\prime },T_{d}{}^{\prime }\right)$$

The process of information gain thus ends up in the middle of the controversial subject of the correct relativistic transformation of temperatures [15,16,17,18,19,20]. An equality sign in Equation (25) only applies in case detector temperatures transform in the same manner as the particle energies. This equality, which assumes equal significances independent of the frame of reference, implies that moving objects appear to be hotter than stationary ones as predicted by the Ott transformation [16]. These final considerations suggest that measuring the significance of observable events in different frames of reference could provide experimental tests to shed more light on the controversial subject of relativistic temperature transformations.

## Figures and Tables

**Table 1:** Molecular entropies relating to molecular rotations, vibrations and electronic excitations as derived from the simplified models of molecular rotators, vibrators and electronic two-level systems [13].

Model	Molecular Entropy
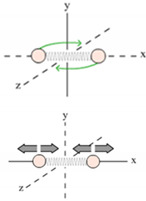 Molecular rotator Molecular vibrator	$s_{BE}\left(\omega ,T\right)=k_{B}\left[\frac{\left(\frac{\hbar \omega }{k_{B}T}\right)exp\left(-\frac{\hbar \omega }{k_{B}T}\right)}{1-exp\left(-\frac{\hbar \omega }{k_{B}T}\right)}-ln\left[1-exp\left(-\frac{\hbar \omega }{k_{B}T}\right)\right]\right]$
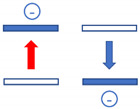 Electronic two-level system	$s_{FD}\left(E_{el},T\right)=k_{B}\left[\frac{\left(\frac{E_{el}}{k_{B}T}\right)exp\left(-\frac{E_{el}}{k_{B}T}\right)}{1+exp\left(-\frac{E_{el}}{k_{B}T}\right)}+ln\left[1+exp\left(-\frac{E_{el}}{k_{B}T}\right)\right]\right]$

**Figure A1:**
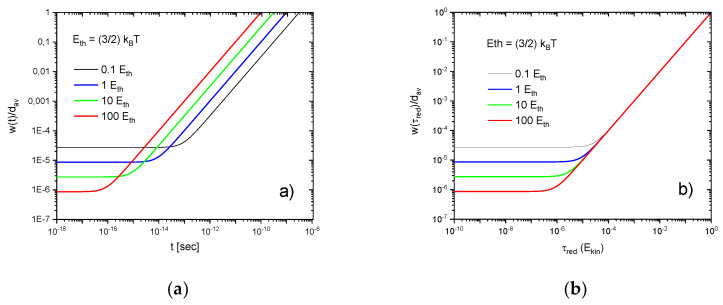
Wave-packet widths w(t) relative to the average mean-free path $d_{av}$ as a function of time (**a**) and reduced time $\tau =t/\tau_{coll}\left(E_{kin}\right)$ (**b**) after a gas-kinetic collision has occurred. The individual curves denote solutions for increasing molecular kinetic energies in units of Eth=33kBT. Molecular parameters correspond to normal air (N_2_, O_2_).

## Appendix A

In order to arrive at the time dependence of $i_{loc}\left(t\right)$, the functional form of the quantum volumes $V_{Q}\left(E_{kin},t\right)$ needs to be obtained. As quantum volumes are the 3d analogs of 1d Schrödinger wave packets, their time evolution can be studied by referring to the textbook example of the 1d Schrödinger wave dispersion [21]. Immediately after collision the extension of a 1d wave packet can be approximated by a Gaussian probability distribution ρ(0) of width:

(A1) $$w_{0}\left(E_{kin}\right)=\frac{\hbar }{\sqrt{2ME_{kin}}}$$

Rearranging it is found that the initial wave packet width conforms to the position-momentum uncertainty relationship:

(A2) $$\sqrt{2ME_{kin}}w_{0}\left(E_{kin}\right)=p\left(E_{kin}\right)w_{0}\left(E_{kin}\right)=\hbar$$

At times t>0 the distribution ρ(x) evolves with time according to

(A3) $$\rho \left(x\right)=\frac{1}{\sqrt{\pi }w\left(E_{kin},t\right)}exp\left[-\frac{{\left(x_{I}-vt\right)}^{2}}{w{\left(E_{kin},t\right)}^{2}}\right]$$

with $x_{I}$ denoting the site of impact, $v=\sqrt{2E_{kin}/M}$ the speed of the outgoing particle and t the time after impact. As this wave packet travels, its width will continually increase according to:

(A4) $$w\left(E_{kin},t\right)=w_{0}\left(E_{kin}\right)\sqrt{1+{\left(\frac{\hbar t}{Mw_{0}{\left(E_{kin}\right)}^{2}}\right)}^{2}}$$

Values of $\Delta x\left(E_{kin},t\right)$ are shown in Figure A1a as a function of time for a range of values of $E_{kin}$. Approximations for $\Delta x\left(E_{kin},t\right)$ for small and large times are:

(A5) $$w\left(E_{kin},0\right)=\frac{\hbar }{\sqrt{2ME_{kin}}};\;t<\tau_{ret},$$

(A6) $$w\left(E_{kin},t\right)=\sqrt{\frac{2E_{kin}}{M}}t;\;t>\tau_{ret}.$$

From these extrapolations the retention time, $\tau_{ret}$, can be obtained, below which the wave packet extension remains close to its initial value $w\left(E_{kin},0\right)$:

(A7) $$\tau_{ret}\left(E_{kin}\right)=\frac{\hbar }{2E_{kin}}$$

Rearranging, it is found that the kinetic particle energy and the retention time conform to the time-energy uncertainty relation:

(A8) $$E_{kin}\tau_{ret}=\frac{\hbar }{2}$$

Further following the growth of wave packet width in Figure A1a, it can be seen that initially more localized wave packets will reach linear dimensions comparable to the average mean-free distance $d_{av}$ earlier than less localized ones. As high-energy particles traverse distances of $d_{av}$ in shorter times than low-energy ones, the mean-free time between successive collisions emerges as:

(A9) $$\tau_{coll}\left(E_{kin}\right)=\frac{1}{N\sigma_{coll}}\sqrt{\frac{M}{2E_{kin}}}=d_{av}\sqrt{\frac{M}{2E_{kin}}}$$

With this result the wave packet width can be evaluated after which a molecule has travelled through a distance $x=d_{av}$:

(A10) $$w\left(E_{kin},\tau_{coll}\right)=\frac{1}{N\sigma_{coll}}=d_{av};\;\sigma_{coll}=\mathrm{collision}\;\mathrm{cross}\;\mathrm{section}$$

This latter result shows that independent of the kinetic energy, the particle wave functions always reach widths comparable to the average mean free distance $d_{av}$ before undergoing follow-on collisions. This scaling of the wave packet widths with the reduced time $\tau =t/\tau_{coll\;}\left(E_{kin}\right)$ is illustrated in Figure A1b.

With the results for $\Delta x\left(E_{kin},t\right)$ (Equation (A4)), the energy-dependent size of the 3d quantum volumes can be estimated:

(A11) $$V_{Q}\left(E_{kin},t\right)={\left(w\left(E_{kin},t\right)\right)}^{3}.$$

With this the information can be obtained which is missing with regard to the location of molecules moving within their own mean-free volumes $V_{mol}$:

(A12) $$i_{loc}\left(E_{kin},t\right)=\frac{1}{ln\left(2\right)}\left[\frac{V_{mol}}{V_{Q}\left(E_{kin},t\right)}\right]$$
